# London's Churches, Chapels, and People

**Published:** 1887-12-17

**Authors:** 


### The Hospital

A Weekly Institutional Journal of

Science, Medicine, and Philanthropy.

*For Hospitals, Asylums, Medical Practitioners, Students, Nurses, Families, Parishes, Congregations, and the Charitable Public.*

Vol. III. No. 64.] [December 17, 1887.

### London's Churches, Chapels, and People.

The year of Queen Victoria's Jubilee will be famous for many things, and there are hopeful signs that it will be for ever remembered as the year in which the inhabitants of the greatest city in the Empire aroused themselves to manfully face and remove the distress which has so long hung like a pall over London. As the last days of 1887 pass rapidly away, evidence accumulates that at last the public conscience and with it the energies of the inabitants of London is being moved to a sense of its duty and responsibilities. Heretofore the greatest minds and the most noble-hearted of England's sons and daughters have recoiled from facing the metropolitan distress as a whole, being appalled by the size of the town and the gigantic character of the task. The reason for the dawning of brighter days for London's poor is not far to seek. The Registration Act, which divided London into sixty single-member constituencies, has done more to produce this result than anything else. People who felt it to be hopeless to attempt to deal with the distress existing amongst four million souls have taken heart of grace, and now realise that sixty constituencies, with an average population of less than 70,000 each, are individually capable of being dealt with effectually and well. Of course, there is not equal activity throughout the sixty Parliamentary divisions of London, but the more active spirits among the metropolitan members, inspired by the example of Lord Randolph Churchill in Paddington, are arousing themselves to action, which cannot fail ultimately to lead to the best results. In The Hospital for the 3rd inst. we showed the present condition of London as regards its charities, its Poor-law system, and the works which might be undertaken eventually with the certainty that they would provide sufficient employment for the whole of the unemployed poor of London for ten years to come. To-day we give elsewhere the outline of a scheme which will provide a permanent solution for this question in every constituency which decides to adopt it. The first step to take to secure this desirable result rests with the gentlemen who represent each of the sixty constituences in the House of Commons. If every metropolitan member will call together the leaders of all parties, classes, and creeds within his constituency, for the purpose of ascertaining the extent of the distress within its boundaries to-day, the nature and amount of the relief agencies at work, and to whom that relief is given, and the public works which can be commenced forthwith to provide employment for the deserving poor, then a solution will be found, and all classes in London may congratulate themselves on the accomplishment of a noble and necessary work. The people of all classes in every constituency are eager to face this question promptly, and the responsibility of leaving the distress in any Parliamentary division of the metropolis undealt with and unrelieved must lie at the door of each metropolitan member who fails to summon a conference forthwith. The people cannot act without leaders. Each metropolitan constituency looks to its member, or, in his absence, to the chairman of his committee, to lead the inhabitants to a wise solution of a vast and difficult problem. We make bold to say, and we believe it will be found to be true, that should any Parliamentary representative show by his inaction that he is careless or thoughtless for the needs of the poor and blind to the necessity of the present situation, he will be compelled to give place to one who has a keener and better sense of the responsibilities attaching to those who represent any section of the metropolitan public in Parliament.

But what are the churches and chapels doing in this matter? Are they prepared to leave the work of facing the distress in each constituency as a whole, as distinct from the distress in a particular parish, to others, or will they co-operate and combine to secure an adequate and permanent settlement once and for all? We believe no uncertain answer will be given by the clergy, ministers, and congregations to this question. The summoning of the representative inhabitants of all classes and creeds by Lord Randolph Churchill in Paddington has brought to light a fact which is not recognised, because it is not generally known. It appears that very many parishes are so well and systematically organised by the clergy that all the poor within the parochial boundaries are known, and a precise and accurate statement can be given, when asked for, of the circumstances and needs of families and particular individuals. No doubt this state of affairs is largely due to the establishment of the Bishop of London's Fund, which has enabled new churches to be built and new parishes to be formed, in sufficient numbers to make the population entrusted to the majority of the clergy assume limits so reasonable as to be fairly manageable. In the parishes organised on the system we have referred to, it is asserted, and having put the assertion to the test we believe it to be precisely true, that strangers who come into the parish—that is, the fluctuating and predatory population of a great city like London—are recognised on their arrival, systematically visited, and looked after, to an extent as surprising as it is satisfactory. Many people are found always ready to scoff at religious organisations, and to complain of the inefficiency of the clergy and ministers of religion. It is therefore with supreme satisfaction that we are able to-day to publish an account of the organisations existing in St. Paul's, Harrow Road, and in the districts under the charge of the Roman Catholic Priest and the Rev. Dr. Clifford, the able minister of the Westbourne Park Chapel. We believe that a similar system is followed in St. Peter's, Eaton Square, and we doubt not in other metropolitan parishes. Honest scoffers who read these accounts will be silenced and convinced by this evidence of the thoroughness with which religious organisations carry out their work in London to-day. We have merely selected the two instances referred to as typical of many similar ones in various parts of the metropolis, and we will gladly send our Special Commissioner to visit any parish or district which has been organised under any clergyman or minister, on receiving an invitation from those interested. Here, at any rate, is one surprising and excellent result of the attempts already made to arouse and fix public attention, not on the necessity of dealing with the distress, but on the practicability of finding a wise solution, if the people, under the leadership of their members in each constituency, will quit themselves like men.

A year ago, six months ago, and even to-day, many people who have never seriously studied this question, or who have no accurate or detailed knowledge of the facts, did and do laugh and shake their heads at the bare idea of attempting to attain precise and accurate information of the needs and circumstances of every poor family and person resident in each metropolitan constituency at the present time, and henceforward for all time. Yet these same people, if they will only devote the necessary time to the perusal of the scheme we give elsewhere, for organising each constituency, together with the facts stated concerning particular parishes and congregational districts, must confess, if they are candid and true men and women, that the task is not only not hopeless, but relatively simple. We show that a conference of all classes, having assembled under the chairmanship of the M.P. for the district, will be able at once to establish a system which shall provide every family or person who has fallen into want with the two things needful: First, relief from pressing wants—food, if hungry; fuel, if cold; or clothing, if naked. This is the work of relief. Secondly, a long, steady, patient pull by a wise, strong hand up on to solid ground. This is the work of the voluntary visitors, and it will prove helpful not only to the poor to whom they minister, but to the many well-to-do people who reside in London for the greater part of each year, many of whom desire to find an opening for their energies and employment for their leisure.

Of course, as we have pointed out elsewhere, everything cannot be done in a moment, and if the conference in each metropolitan constituency succeeds during the present winter in collecting, arranging, and registering the necessary information concerning (*a*) the relief given by the Poor-law authorities and the various charities; (*b*) all the men and women who are out of work, and who desire and deserve to secure it; and (*c*), the establishment of a fund raised by public subscription to defray the necessary expenses, and to provide the necessary relief —and this must be done, to secure the end all desire to reach—when the winter is over, steps can be taken to further develop, extend, and consolidate the machinery thus set in motion in each constituency, so that by October, 1888, every district of London could have the distress within its boundaries ascertained, its amount and requirements precisely recorded, and proposals for its effectual and permanent relief ready for discussion and adoption. As we said last week, what the poor in London require at the present time is not so much alms as a friend. Under the old, bad system, before the passing of the Registration Act, each poor man or woman was lost in the multitude of inhabitants resident within this vast city. Now, under the system here advocated, every poor man will be recognised as belonging to a defined district or place, just as if he lived in a provincial town. The effect of the recognition of this fact by the poor and by their richer neighbours, will be to kindle throughout London a feeling of brotherliness which can only exist where there is actual personal knowledge between poor and rich—between those who live in the courts and alleys and those who occupy houses in the terraces and squares. To reside in London will then be, for a poor man, a less dreary task than at present, because he will be able henceforward to live rather than to exist. One of the delights of London has heretofore been felt by many people to be the absence of all knowledge concerning one's neighbours. There is no doubt a certain pleasure to be found by some in the solitude of the great city. But the parable of the good Samaritan is as applicable to London as it was to the highway in the days of our Lord, and the recognition of this fact will move men's hearts and consciences to such action as must ultimately result in a wise, thorough, and permanent solution of this most pressing question.

